# Supplementary material for: Analysis of Intratumoral Heterogeneity in Myelodysplastic Syndromes with Isolated del(5q) Using a Single Cell Approach
Source: Cancers (Basel). 2021 Feb 17;13(4):841. doi: 10.3390/cancers13040841 (PMC7922695; doi:10.3390/cancers13040841)
Supplement: Supplementary file 1 [file cancers-13-00841-s001.pdf]

Supplementary Materials:

Analysis of Intratumoral Heterogeneity in Myelodysplastic Syndromes with Isolated del(5q) Using a Single Cell Approach

Pamela Acha, Laura Palomo, Francisco Fuster-Tormo, Blanca Xicoy, Mar Mallo, Ana Manzanares, Javier Grau, Silvia Marcé, Isabel Granada, Marta Rodríguez-Luaces, María Diez-Campelo, Lurdes Zamora and Francesc Solé

Supplementary Methods

Fluorescence activated cell sorting of $CD34^{+}CD117^{+}CD45^{+}CD19^{-}$ HSPC

Bone marrow samples from each patient were used to isolate $CD34^{+}CD117^{+}CD45^{+}CD19^{-}$ HSPC using fluorescence activated cell sorting (FACS). Staining was performed using CD34, CD117, CD45 and CD19 antibodies (BD Biosciences, San José, CA, USA). After 15 minutes incubation, cells were washed once with phosphate-buffered saline (PBS) and finally suspended in PBS at a final concentration of 10×10^{6} cells/mL (minimum volume of 1 mL).

Once sorting was performed using a FACSAria™ II (BD Biosciences), cells were cryopreserved (20% DMSO, 70% FBS). At least 3 cryovials of 30.000 cells/100μL were stored for further SC experiments.

WES analysis

Sequencing reads were mapped to the human reference genome (hg19/GRCh37) using GEM software [1]. Somatic variant calling was performed using MuTect, Strelka2 and Lancet [2–4], from data of both BM tumoral samples and paired T-CD3+ lymphocytes germline control samples. Variant candidates were obtained and then annotated using population databases (dbSNP, ExAC, Exome Variant Server y 1000 Genomes), clinical databases (COSMIC, ClinVar) and in silico algorithms (SIFT, PolyPhen2, Mutation Assessor, Mutation Taster).

Variants located in highly variable regions or with low coverage were discarded. Then, variants were filtered according to the variant allele frequency (VAF): variants with VAF >5% were considered, as well as variants with VAF≤5% and at least 25 altered reads for those variants that have been previously reported in hematological neoplasms.

Four to six candidate mutations, with the previously mentioned criteria, were selected per patient for subsequent SC analysis.

SNP-A analysis

Data were analyzed using the Chromosome Analysis Suite version 3.0.0.42 (Thermo Fisher Scientific), using annotations of genome version NCBLv33.1 (hg 19). Paired sample analysis with T-CD3+ lymphocytes DNA was used to rule out germline lesions. Software-reported copy number alterations of 50 Kb that carried a minimum of 10 aberrant probes were considered. A visual analysis was also performed.

For copy number neutral-loss of heterozygosity (CNN-LOH), ≥50 altered probe sets were required and at least 2 Mb and 25 Mb in size for telomeric and interstitial CNN-LOH, respectively.

SC capture

For each studied patient, two types of cells were isolated using this protocol:

$CD34^{+}CD117^{+}CD45^{+}CD19^{-}$ BM-HSPC from each studied moment (DX/PRE and available FU samples).

T-CD3+ lymphocytes from a healthy donor. These cells served as wild type and normal copy number control.

Previously mentioned cells were thawed and then resuspended using suspension buffer (Hanks´ Balanced Salt Solution [Gibco, 14175-053] supplemented with 1mM EDTA [Sigma-Aldrich, E6758] and 2% of fetal bovine serum [Gibco, 10270106]). Then, cells were incubated at 37°C for 20 minutes.

A volume of 15 μL at 1000 cells/μL was loaded into the corresponding Single-Cell Open App chip and loading program was run in Fluidigm C1 instrument. In this step, each cell is isolated in one of the 96 individual capture sites. Then, visual inspection using an optical microscope was performed in order to annotate capture sites containing only one cell and excluding, for further analysis, those having more than one cell or those empty capture sites.

Next steps in Fluidigm C1 are cell lysis and targeted DNA pre-amplification. Reaction mixes are loaded and reactions take place in each capture site. Cell lysis mix consists on 0.3 mg/mL of proteinase K (reference 19131, Qiagen, Hilden, Germany) and 0.01% of Tween 20 (reference P9416, Sigma Aldrich, Saint Louis, MI, USA) in HEPES buffer

(reference 51558, Sigma Aldrich). Incubation takes place for 50 minutes at 60°C, followed by 10 minutes at 98°C. Pre-amplification master mix is prepared with 32.5 µL of TaqMan™ PreAmp Master Mix 2X (Thermo Fisher Scientific) and 6.5 µL of a 1:100 primer mix dilution (0.18 µM of each mutation/CNA specific assay, see Supplementary Tables S2-S4). DNA denaturation takes place for 15 minutes at 95°C followed by 18 cycles of amplification: 25 seconds at 95°C followed by 4 minutes at 60°C.

Finally, pre-amplified DNA of each single cell is recovered and 1:5 diluted with DNA suspension buffer (reference 100-5319, Fluidigm). Dilutions are stored in 96 well-plates at -20°C.

Supplementary Tables

Table S1. Genes with covered exons in the custom myeloid panel.

Gene	Exons	Gene	Exons	Gene	Exons	Gene	Exons
ASXL1	9, 11-12	*DNMT3A*	All	*KMT2A*	3-9	*SF3B1*	10-16
ASXL2	11-12	*EZH2*	All	*KRAS*	2-3	*SMC3*	All
BCOR	All	*ETV6*	All	*MPL*	All	*SRSF2*	1
BCORL1	All	*FLT3*	13-15, 20	*NF1*	All	*STAG1*	All
BRAF	15	*GATA1*	All	*NPM1*	10-11	*STAG2*	All
CALR	9	*GATA2*	All	*NRAS*	2-3	*TET2*	All
CBL	8,9	*IDH1*	All	*PTPN11*	All	*TP53*	3-11
CEBPA	All	*IDH2*	All	*RAD21*	All	*U2AF1*	2-6
CSF3R	All	*JAK2*	All	*RUNX1*	All	*WT1*	2-10
CSNK1A1	3-4	*KIT*	2,8-14,17	*SETBP1*	4	*ZRSR2*	All

Table S2. Annotation of variants detected by WES and TDS in the studied samples.

UPN	Gene	Chr	Start position (hg19)	Reference allele	Alternate allele	dbSNP ID	COSMIC ID	Minor allele frequency (gnomAD)	SIFT Prediction	SIFT Score	Polyphen2 Prediction	Polyphen2 Score
P1	*CUX1*	7	101847749	C	T	NA	NA	NA	NA	NA	NA	NA
P1	*SETBP1*	18	42531917	T	C	rs267607038	NA	1,76E-05	Deleterious	0.0	Probably Damaging	1.0
P1	*MAP7D2*	X	20134882	T	C	NA	NA	NA	Tolerated	0.327	Benign	0.075
P1	*TENM1*	X	123615653	C	A	NA	NA	NA	NA	NA	Probably Damaging	1.0
P1	*LRTOMT*	11	71819886	G	A	rs963049398	NA	1,84E-04	Tolerated	0.173	Benign	0.006
P1	*CCDC168*	13	103389038	T	G	NA	NA	NA	NA	NA	NA	NA
P1	*TP53*	17	7577094	G	A	rs28934574	COSM10704	8,79E-06	Deleterious	0.002	Probably Damaging	1.0
P1	*NUP93*	16	56862936	C	A	NA	NA	NA	Deleterious	0.001	Probably Damaging	0.995
P1	*UNC79*	14	94158291	T	A	NA	NA	NA	Deleterious	0.049	NA	NA
P2	*LRRC45*	17	79988196	A	G	NA	NA	NA	Deleterious	0.031	Benign	0.361
P2	*CRIPAK*	4	1388324	A	C	rs74377230	COSM1427134	NA	Deleterious	0.0	NA	NA
P2	*CACHD1*	1	65113641	G	T	rs746548532	NA	8,80E-06	Deleterious	0.044	NA	NA
P2	*IL21R*	16	27448835	A	G	NA	NA	NA	Tolerated	0.068	Benign	0.05
P2	*SF3B1*	2	198266834	T	C	rs559063155	COSM84677	0.0001161	Deleterious	0.004	Probably Damaging	1.0
P2	*YLPM1*	14	75266087	C	T	NA	NA	NA	NA	NA	NA	NA
P3	*CGNL1*	15	57836776	C	A	NA	NA	NA	Deleterious	0.001	Probably Damaging	0.992
P3	*IBSP*	4	88727321	G	T	NA	NA	NA	Deleterious	0.03	Probably Damaging	0.999
P3	*SLC22A12*	11	64359260	C	T	NA	NA	NA	NA	NA	Possibly Damaging	1.0
P3	*TRIM24*	7	138145556	A	G	NA	NA	NA	Tolerated	0.146	Probably Damaging	0.987
P3	*SETD2*	3	47103749	T	C	NA	NA	NA	Deleterious	0.002	Possibly Damaging	0.799
P3	*FAT1*	4	187629475	C	T	rs765412973	NA	3,27E-05	Tolerated	0.136	Possibly Damaging	0.644
P3	*TCHH*	1	152081923	T	C	rs375240460	NA	8,87E-06	Tolerated	0.288	Benign	0.107
P3	*PPM1D*	17	58740529	C	A	rs146477590	COSM98068	5,44E-05	NA	NA	NA	NA
P3	*SS18L1*	20	60738552	A	G	NA	NA	NA	Deleterious	0.004	Benign	0.278
P4	*TP53*	17	7578530	A	G	rs267605077	COSM44654	NA	Deleterious	0.002	Possibly Damaging	0.994
P4	*SCUBE1*	22	43614452	G	A	rs762401550	NA	5,44E-05	Tolerated	0.25	Benign	0.0
P4	*BMP7*	20	55758828	C	T	rs749169949	NA	1,63E-04	Deleterious	0.027	Probably Damaging	1.0
P4	*NUP85*	17	73221814	G	C	NA	NA	NA	Deleterious	0.03	Possibly Damaging	0.723
P4	*DNMT3B*	20	31388640	G	T	NA	NA	NA	NA	NA	NA	NA

Abbreviations: chr: chromosome; NA: not available; TDS: targeted deep sequencing; UPN: unique patient number; WES: whole exome sequencing. Information was gathered using myvariant.info [5].

Table S3. Associated biological process and molecular function of the genes in which variants were detected using WES and TDS.

UPN	Gene	Biological process	Molecular function
P1	*CUX1*	Negative regulation of transcription by RNA polymerase II	RNA polymerase II transcription regulatory region sequence-specific DNA binding
P1	*SETBP1*	NA	DNA binding, protein binding
P1	*MAP7D2*	Microtubule cytoskeleton organization	NA
P1	*TENM1*	Regulation of transcription by RNA polymerase III	Protein heterodimerization activity, cell adhesion molecule binding.
P1	*LRTOMT*	Methylation, developmental process	O-methyltransferase activity
P1	*CCDC168*	NA	NA
P1	*TP53*	Regulation of intrinsic apoptotic signaling pathway by p53 class mediator	Transcription regulatory region sequence-specific DNA binding, MDM2/MDM4 family protein binding
P1	*NUP93*	Regulation of glycolytic process, SMAD protein signal transduction, regulation of gene silencing by miRNA	Protein binding, structural constituent of nuclear pore
P1	*UNC79*	Ion transmembrane transport	NA
P2	*LRRC45*	NA	Protein binding
P2	*CRIPAK*	NA	NA
P2	*CACHD1*	Calcium ion transmembrane transport	Voltage-gated calcium channel activity
P2	*IL21R*	Natural killer cell activation, interleukin-21-mediated signaling pathway	Interleukin-21 receptor activity, transmembrane signaling receptor activity, cytokine receptor activity
P2	*SF3B1*	Spliceosomal complex assembly, positive regulation of gene expression	RNA binding
P2	*YLPM1*	Regulation of telomere maintenance	RNA binding
P3	*CGNL1*	Negative regulation of small GTPase mediated signal transduction	Motor activity;protein binding
P3	*IBSP*	Osteoblast differentiation, cell adhesión	Integrin binding
P3	*SLC22A12*	Organic anion transport	Urate transmembrane transporter activity, urate transmembrane transporter activity, PDZ domain binding
P3	*TRIM24*	Negative regulation of cell population proliferation, protein ubiquitination.	p53 binding, chromatin binding
P3	*SETD2*	Angiogenesis, transcription elongation from RNA polymerase II promoter	Histone-lysine N-methyltransferase activity
P3	*FAT1*	Actin filament organization, cell adhesion	Calcium ion binding
P3	*TCHH*	Intermediate filament organization	Calcium ion binding
P3	*PPM1D*	Negative regulation of cell proliferation, DNA damage response, signal transduction by p53 class mediator	Protein serine/threonine kinase activity
P3	*SS18L1*	Chromatin organization, positive regulation of transcription by RNA polymerase II	Transcription coactivator activity
P4	*TP53*	Regulation of intrinsic apoptotic signaling pathway by p53 class mediator	Transcription regulatory region sequence-specific DNA binding, MDM2/MDM4 family protein binding
P4	*SCUBE1*	Inflammatory response, endothelial cell differentiation	Calcium ion binding
P4	*BMP7*	Skeletal system development, epithelial to mesenchymal transition, BMP signaling pathway, epithelial cell differentiation, negative regulation of NF-kappaB transcription factor activity	Cytokine activity, growth factor activity, BMP receptor binding
P4	*NUP85*	Regulation of glycolytic process	Structural constituent of nuclear pore
P4	*DNMT3B*	Negative regulation of transcription by RNA polymerase II, response to hypoxia, DNA methylation	DNA-methyltransferase activity, histone deacetylase binding

Abbreviations: NA: not available; TDS: targeted deep sequencing; UPN: unique patient number; WES: whole exome sequencing. Information was gathered using mygene.info [5].

Table S4. CNA detected in each patient using SNP-A analysis.

UPN	Chr	Cytoband		Coordinates		Copy number	Alteration	Size (Mb)
		Start	End	Start	End			
**P1**	**5**	**q14.3**	**q34**	89575437	163450743	1	Loss	73.88
P2	5	q21.2	q34	102986653	162755919	1	Loss	59.77
	X	p22.31	p22.31	6449753	8135644	1	Loss	1.67
P3	5	q14.3	q34	86255729	166126310	1	Loss	79.87
P4	5	q21.3	q34	107937392	165840296	1	Loss	57.90

Abbreviations: Chr: chromosome; Mb: megabases; UPN: UPN: unique patient number; SNP-A: single nucleotide polymorphism arrays

Table S5. Specific primers and assays designed for selected SNV for each patient included in the study.

UPN	Gene	Mutation	Forward primer	Reverse primer	Mutant Reporter (FAM-NFQ)	Wild-type Reporter (VIC-NFQ)
P1	*NUP93*	p.Ala158Asp	ACCCTTGTGACTGTCTTTGGAAATT	TCAGGAAACTTCGAACCAATTGGTAA	CCAGCTGGTCCTGATG	CAGCTGGGCCTGATG
	TP53	p.Arg150Trp	CTTTCTTGCGGAGATTCTCTTCCT	GCTTTGAGGTGCGTGTTTGTG	TGCGCCAGTCTCT	TGCGCCGGTCTCT
	LRTOMT	p.Arg224His	CCGCTTCTTGCAGTATGCTAAG	GATGGCAGGGAAGTCTGGAA	CGCTACCACTGCCG	CGCTACCGCTGCCG
	SETBP1	p.Ile871Thr	TCCCACAGTGAGGAGACGAT	CTTGGTCAGAAGTGCTGTTGTTG	CTGTCCCAGTGCCGC	TCTGTCCCAATGCCGC
	CUX1	p.Arg1007*	CGAAGCCATGGAGCAAGCT	CAGAGCTGCATCCGGATGA	AGGGTTCTCAGCCTTT	AGGGTTCTCGGCCTTT
P2	*SF3B1*	p.Lys700Glu	TGGCCAAAGCACTGATGGT	TGGTTTTGTAGGTCTTGTGGATGAG	AGCAGGAAGTTCG	CAGCAGAAAGTTCG
	YLMP1	p.Arg1363*	GGAGAGAAGAAAGAAATCGAGAGCAT	TCGAATCCTCAACTCACCCCTAT	ACGGAAATCTCAATCATA	CACGGAAATCTCGATCATA
	CACHD1	p.Arg387Ser	CATGATGGTGCTGAATCAGTTGAG	ATCCGGTTGGGAAGGTTTGT	TGGGCAGTTTCTAC	TGGGCAGGTTCTAC
P3	*PPM1D*	p.Cys478*	CCCTCAAAAGATCCAGAACCACTTG	CCAATTGGAAGGCTATTATTCAAAGAATCA	CTTTAGCTCAATTTTC	TTAGCGCAATTTTC
	SETD2	p.Asp2066Gly	GGAGCTTCTTCGTTTCCTTTTCTCT	CCCGAAGACTCCTAATAGGTCAAG	CCCAGGCAAGCAA	AGACCCAGACAAGCAA
	SS18L1	p.Ser202Gly	GTCGTTCAGTCTCCATGATGCA	CGACTGGCCCTGGTAGTG	CGCACTACGGCTCG	TCGCACTACAGCTCG
	FAT1	p.Ala503Thr	CAATCGCAAACGGCACATGAT	GTGAGAACGGGTACGTGACAT	ACAGTATCACAAATTT	CAGTATCGCAAATTT
	SLC22A12	p.Pro78Ser	TGAGGCCCTCCTGGCTATTT	GAAGCGGCGGCACTG	CCCGGCGAGATGG	CCCGGCGGGATGG
	TRIM24	p.Tyr88Cys	CCCTGCCTGCACTCTTTCTG	GGCCGAGCCCAGCAT	CAGCGCTGCCTCAT	CCAGCGCTACCTCAT
P4	*TP53*	p.Phe95Leu	GTGGAATCAACCCACAGC	AACTCTGTCTCCTTCCTCTTC	AGATGCTTTGCC	CAAGATGTTTTGCCA
	NUP85	p.Ala293Pro	AGAGTTCAGCACAAATGTCTCT	GTACAAGAGCCGAGTCACT	AAGCTCCCTTG	AAGCTGCCTTG
	BMP7	p.Arg303His	CTAAGCATACCTGCCACG	TCCACTTCCGCAGCAT	TGGAGTGGTTC	TGGAGCGGTTC

Patient P1, P2 and P3 assays are from TaqMan (Thermo Fisher Scientific) while P4 assay are LNA prime time probes (Integrated DNA Technologies). Abbreviations: SNV: single nucleotide variant. UPN: unique patient number

Table S6. Pre-designed TaqMan CNA.

UPN	Gene Symbol	Chromosome	Cytogenetic band	Assay ID	Context sequence
Reference CNA	*B2M*	15	15q21.1	Hs00119644_cn	TGTTTTTGTTTCACTGTCCTGAGGA
				Hs03900880_cn	CCAGAGCAAACTGGGCGGCATGGGC
				Hs04450638_cn	TGATTGCCTCTGAAGGTCTATTTTC
P1, P2, P3 and P4	*RPS14*	5	5q33.1	Hs03575527_cn	CGCCACAGGAGAGAAGCCCAAAGTT
				Hs06120504_cn	CTTTCCAGAAAGGGAAACAGGCTGA
				Hs03542482_cn	TTCTTACCTGGACAAAACAGCATTT
P2	*STS*	X	Xp22.31	Hs05600643_cn	GGCTACTTTGCAGACGGGGAAGCTG
				Hs05690005_cn	GCTTCTGTGTGTCCTCGGATGCAGC
				Hs05669475_cn	TGCATGGAAGTGCTCACCTGATTCA

Abbreviations: CNA: copy number assay. UPN: unique patient number.

Table S7. Reference genotyping assay, used in all tested samples.

Assay name	rs346172	Location	Intronic variant	Chromosome	19	Position	13843233

Forward Primer	Reverse Primer	Wild-type Reporter (VIC-NFQ)	Variant Reporter (FAM-NFQ)
CAAACGCCATCCGGACCA-TA	GCATCTTT-GAGCTCCTAAGTCCAA	CACTATCGCCATCACTTT	ACTATCGCCGTCACTTT

Table S8. PCR cycling conditions in the BioMark HD.

Stage	Temperature	Time
Thermal Phase	70°C	40 minutes
	60°C	30 seconds
Hot start phase	98°C	1 minute
Amplification (35 cycles)	97°C	20 seconds
	60°C	20 seconds

References

1. Marco-Sola, S.; Sammeth, M.; Guigó, R.; Ribeca, P. The GEM Mapper: Fast, Accurate and Versatile Alignment by Filtration. *Nat. Methods* **2012**, *9*, 1185–1188, doi:10.1038/nmeth.2221.
2. Cibulskis, K.; Lawrence, M.S.; Carter, S.L.; Sivachenko, A.; Jaffe, D.; Sougnez, C.; Gabriel, S.; Meyerson, M.; Lander, E.S.; Getz, G. Sensitive Detection of Somatic Point Mutations in Impure and Heterogeneous Cancer Samples. *Nat. Biotechnol.* **2013**, *31*, 213–219, doi:10.1038/nbt.2514.
3. Kim, S.; Scheffler, K.; Halpern, A.L.; Bekritsky, M.A.; Noh, E.; Källberg, M.; Chen, X.; Kim, Y.; Beyter, D.; Krusche, P.; et al. Strelka2: Fast and Accurate Calling of Germline and Somatic Variants. *Nat. Methods* **2018**, *15*, 591–594, doi:10.1038/s41592-018-0051-x.
4. Narzisi, G.; Corvelo, A.; Arora, K.; Bergmann, E.A.; Shah, M.; Musunuri, R.; Emde, A.-K.; Robine, N.; Vacic, V.; Zody, M.C. Genome-Wide Somatic Variant Calling Using Localized Colored de Bruijn Graphs. *Commun. Biol.* **2018**, *1*, 20, doi:10.1038/s42003-018-0023-9.
5. Xin J, Mark A, Afrasiabi C, Tsueng G, Juchler M, Gopal N, Stupp GS, Putman TE, Ainscough BJ, Griffith OL, et al. High-performance web services for querying gene and variant annotation. *Genome Biology*. **2016**, 17(1), 1-7, doi: 10.1186/s13059-016-0953-9.
